# Invasive Mucormycosis Induced Pneumopericardium: A Rare Cause of Pneumopericardium in an Immunocompromised Patient

**DOI:** 10.1155/2017/1424618

**Published:** 2017-05-17

**Authors:** Sana Khan, Muhammad Waqar Elahi, Waqas Ullah, Hafez Mohammad Ammar Abdullah, Ejaz Ahmad, Mayar Al Mohajer, Aneela Majeed

**Affiliations:** ^1^Griffin Hospital, Ansonia, CT 06401, USA; ^2^Khyber Teaching Hospital, Peshawar 25000, Pakistan; ^3^Nishtar Hospital, Multan 60000, Pakistan; ^4^University of Arizona, Tucson, AZ 85701, USA

## Abstract

*Mucor* and* Rhizopus* cause life-threatening infections primarily involving the lungs and sinuses, which disseminate very rapidly by necrosis and infarction of the contiguous tissues. We present a case of a 64-year-old African American posttransplant patient who presented with a productive cough and weight loss. He had a past surgical history of renal transplant for renal cell carcinoma and was on dual immunosuppressive therapy, that is, mycophenolate and tacrolimus. During his hospital stay, he developed a pneumopericardium due to the direct extension of a lung lesion. The diagnosis was made by radiological imaging and PCR result which was consistent with* Mucor* species. He was treated with antifungal therapy. The purpose of this report is to highlight the unusual association of mucormycosis with pneumopericardium.

## 1. Introduction

Mucormycosis is caused by a filamentous fungus which belongs to the subphylum mucoromycotina. Diabetics and immunocompromised patients are at a higher risk to acquire the infection but a few cases have been reported in immunocompetent patients with severe soft tissue trauma. It is acquired by inhalation of environmental spores (sporangiospores). Clinical presentation of this disease varies depending upon the involvement of the organ and immune status of the patient. The most common manifestations are rhinocerebral and pulmonary but other manifestations include cutaneous, gastrointestinal, and disseminated infections [1, 2]. A high index of suspicion is essential because the signs and symptoms and the radiological findings are very nonspecific. Diagnosis is usually made by histopathological documentation of ribbon-like hyphae in tissue. Some researchers suggest PCR for a rapid diagnosis to save time [2, 3]. Successful therapy involves early diagnosis, surgical debridement of the infected tissues, high dose systemic antifungal therapy (IV amphotericin B), and reversal of predisposing factors [3, 4]. Untreated cases of mucormycosis have devastating complications. In DKA patients, high activity of ketone reductase enzyme in the acidic and hyperglycemic environment leads to systemic dissemination of the infection [4, 5]. Similarly, in immunocompromised and organ transplant patients, infection from the lungs can spread contiguously to the skin and mediastinum or homogeneously to distant organs. Very rarely the lung infection can spread to the pericardium.

## 2. Case Presentation

A 64-year-old African American male patient presented with a two-week history of a productive cough associated with low-grade fever, worsening dyspnea, generalized weakness, and an unintentional weight loss of 20 lbs over the previous 3 months. He had a past history of bilateral nephrectomy for renal cell carcinoma and had deceased donor renal transplant for end-stage kidney disease three months prior to presentation. He was on hemodialysis for almost 10 years prior to renal transplantation. The patient was on maintenance immunosuppression consisting of mycophenolate mofetil, tacrolimus, and corticosteroids along with trimethoprim/sulfamethoxazole and valganciclovir for microbial prophylaxis. His comorbidities included diabetes mellitus, hypertension, COPD, and chronic rate-controlled atrial fibrillation. He denied any ill contact, travel history, night sweats, hemoptysis, or altered bowel habits.

**Figure 1:**
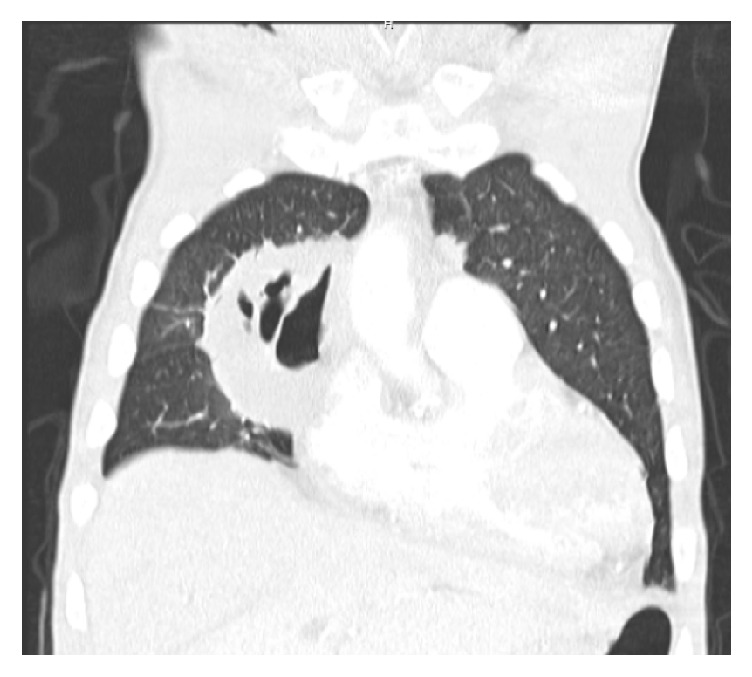
CT of the chest revealing a large right middle lobe cavitary mass (6.5*∗*7.6*∗*8.1 cm^3^) with bilateral pulmonary nodules and a small pericardial effusion.

On examination, he had stable vital signs except for low-grade fever. His chest examination revealed decreased breath sounds in the right upper zone, bilateral basal crackles, and a prominent pericardial rub. Lung examination revealed dull percussion notes in the lower lobes bilaterally. Laboratory data showed a low hemoglobin level of 8.2 g/dL with a mean corpuscular volume of 69 fL/red cell, a leukocytosis of 16,700/*µ*L, and a platelet count of 544,000/*µ*L. His renal function tests were normal with a serum creatinine level of 0.79 mg/dL and blood urea nitrogen of 19 mg/dL. He had normal serum electrolyte levels. His fasting serum glucose level was 163 mg/dL. CT of the chest was done which showed a large right middle lobe cavitary mass (6.5*∗*7.6*∗*8.1 cm^3^) with bilateral pulmonary nodules and a small pericardial effusion (Figure 1).

Blood and sputum cultures were obtained and the patient was initiated on vancomycin and meropenem. Mycophenolate, tacrolimus, and prednisone were held on admission. His white cell count continued to remain elevated and it was decided to biopsy the lung cavitary mass. An ultrasound guided core needle biopsy was attempted but the patient desaturated during the first attempt. He was transferred to intensive care unit due to acute respiratory distress and required mechanical ventilation. After he became hemodynamically stable, a needle biopsy was successfully reattempted. Biopsy report of the lung tissue revealed necrotic lung parenchyma with broad aseptate ribbon-like hyphae consistent with* Mucor* species. The hyphae were identified on hematoxylin and eosin stain and confirmed with Periodic Acid-Schiff (PAS) and Grocott-Gomori's Silver (GMS) special stains. Mucicarmine special stain was negative. Biopsy of the lung tissue was negative for malignant cells. A PCR analysis of histologic specimen was positive for mucormycosis; however, the fungal cultures remained negative. Amphotericin B (lipid formulation) was initiated. Blood cultures were also positive for vancomycin-resistant enterococci (VRE), so daptomycin was started and vancomycin and meropenem were discontinued.

Despite antifungal and antibiotic treatment, the respiratory status of the patient did not improve and he had to be reintubated on hospital day 8. The following day, the patient became hypotensive with a blood pressure of 80/60 mmHg and a heart rate of 110 beats/min. He also developed acute kidney injury with a creatinine of 1.6 mg/dL and anuria of 200 mL over 24 hours. Amphotericin B was switched to isavuconazonium sulfate, due to his rising creatinine levels.

**Figure 2:**
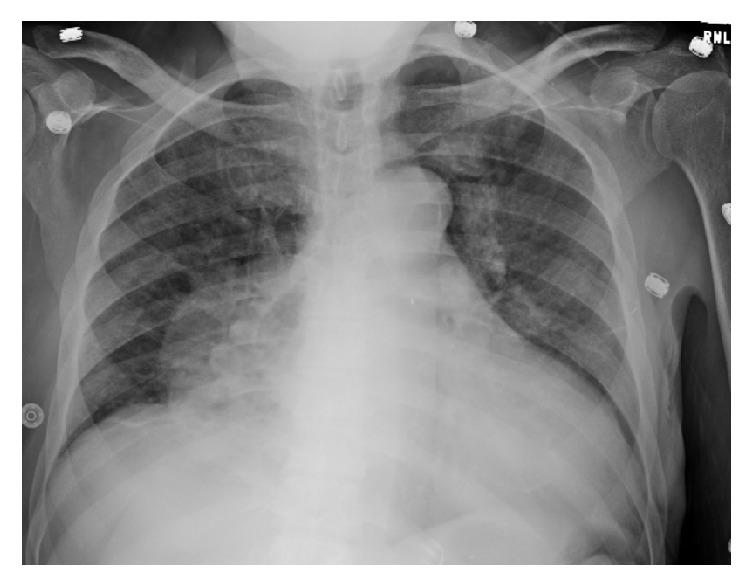
Chest X-ray showing a pneumopericardium, most probably due to the direct extension of the fungal infection (day 15).

**Figure 3:**
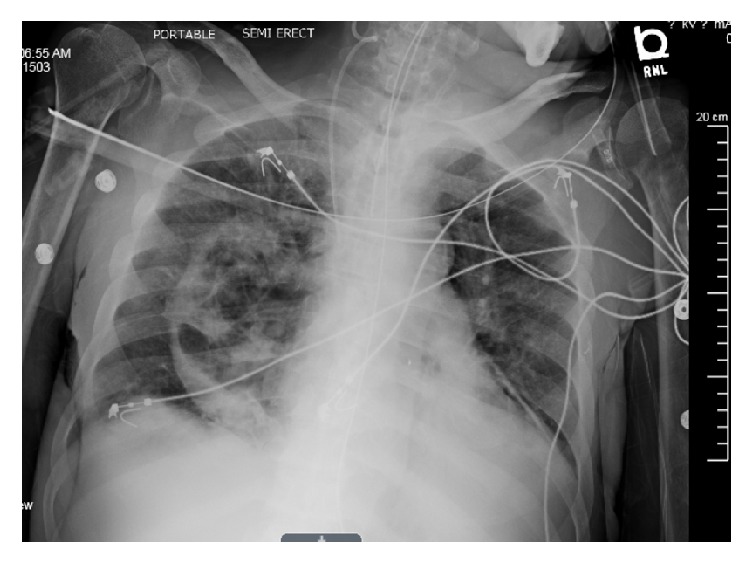
Chest X-ray showing a new patchy right lower lobe opacity consistent with pneumonia and persistent pneumopericardium (day 17).

On hospital day 15, chest X-ray revealed air in the pericardial sac, that is, pneumopericardium, consistent with a direct extension of the known cavitary fungal pneumonia in the chest through the mediastinum into the pericardium (Figure 2). Two days later a repeat chest X-ray suggested a new patchy right lower lobe opacity consistent with pneumonia and persistent pneumopericardium (Figure 3). Despite maximal vasopressor and mechanical ventilatory support, the patient expired on hospital day 22.

## 3. Discussion

Pneumopericardium is a collection of gas in the pericardial cavity. It is most commonly caused by direct trauma to the pericardial layer surrounding the heart or due to the aggressive resuscitation of preterm neonates with positive pressure ventilation [5, 15, 16]. Other causes include stomach or liver fistula, lung inflammations, and pericarditis by gas forming bacteria like clostridium [17]. Infections most commonly associated with pneumopericardium are mycobacterium tuberculosis; however, there have been a few reports of fungal infections [6].

Review of the literature showed that more than 50% of fungal pneumopericardium is caused by aspergillus. There have been reports of* Candida* and* Histoplasma* causing pneumopericardium too [6–14]. However, there have been no previous reports of mucormycosis causing pneumopericardium. Most of these patients were immunocompromised; however, most of the cases had lung infections as the primary focus. With appropriate treatment, many of these patients had a successful recovery with the exception of two patients who expired. The characteristic findings of these cases are given in Table 1.

Fungal pneumopericardium occurs mostly due to direct invasion of the pericardial sac by the fungal hyphae, which can additionally lead to hemoptysis as well. Damage to the alveolar walls due to any cause allows air to travel to the lung hilum and mediastinum and all the way to the pericardial cavity [5, 15]. Symptomatic patients present with precordial pain, hypotension, pulsus paradoxus, shortness of breath, and cyanosis occasionally [5, 15, 18]. On auscultation, a characteristic murmur caused by the heart movement against the air or a loud metallic sound is heard [5, 18]. Depending on the amount of gas in the pericardial cavity, patients can have muffled, distant, or absent heart sounds [5, 16, 18]. In some cases, shifting precordial tympany can be felt on percussion [5, 18].

Laboratory investigations like EKG and radiological imaging can aid in the diagnosis. EKG can be normal or show low voltage QRS complexes and ST segment elevations in associated tamponade and pericarditis, respectively [5, 15]. Chest X-ray and echocardiography are both diagnostic for pneumopericardium [5, 18]. Chest X-ray in the posteroanterior view does not distinguish pneumopericardium from pneumomediastinum. However, in pneumopericardium, serial chest X-rays in left lateral decubitus position show air movement in the pericardial sac between two films while the air column remains stable in pneumomediastinum [5, 15]. Similarly, pneumopericardium has an air column below the level of the aortic arch, forming a halo sign in the pericardial cavity while pneumomediastinum forms a triangular air sign behind the sternum [15, 19]. The cardiac shadow appears larger in usual pneumopericardium while it appears to be smaller in tension pneumopericardium [15, 20]. Echocardiography complements chest X-ray findings by demonstrating air around the heart and also shows any pericardial fluid if present; however, the air in the pericardial cavity does interfere with the assessment of cardiac activity [20].

Other useful tests include a CT chest, which provides details of the air column around the heart, and endoscopy if a fistula or an ulcer is suspected [21]. All these investigations also help to exclude other possible causes of dyspnea and chest pain like pericarditis, pneumonitis, aortic dissection, angina, myocardial infarction, and pulmonary embolism. Definitive tests for underlying mucormycosis include a histopathological examination of the tissue sample, culture, and PCR. Calcofluor white and methenamine silver stains can make a presumptive diagnosis of mucormycosis by demonstrating a characteristic broad, nonseptate hyphae with right-angle branching. Diagnosis can be confirmed by the identification of species in tissue cultures through histopathology. However, culture often yields no growth. Polymerase chain reaction (PCR) based techniques on histologic specimens can be more feasible. Furthermore, serum testing adds in differentiating mucormycosis from other fungal infections.

Small asymptomatic pneumopericardium needs close observation, as it usually resolves spontaneously. Symptomatic patients or those with a high risk of recurrence require immediate treatment with needle aspiration and pericardial tube placement for continuous drainage of the air [5, 15]. Refractory cases require urgent thoracotomy with pericardiotomy along with high flow oxygen as it helps in the resorption of the air from the cavity [15, 22]. Patients also require complete bed rest with head elevation, sedation, and analgesia. It is important to give low positive end-expiratory pressure (PEEP) in mechanically ventilated patients as high pressures can worsen the hypotension and pneumopericardium [16, 22]. Untreated cases can lead to cardiac failure and circulatory collapse [5, 16]. It can be accompanied by pneumothorax, pneumomediastinum, and subcutaneous emphysema which by itself carries a high mortality [5, 16].

## Figures and Tables

**Table 1:** Characteristic findings of previously reported cases of fungal pneumopericardium.

Case number	Author	Age/sex	Underlying condition	Primary disease	Organism	Treatment	Outcome
1	Rosenbaum et al. [6]	41/M	N/A	Mycotic aortic aneurysm	*Histoplasma capsulatum *	N/A	Died
2	Xie et al. [7]	51/M	Invasive pulmonary aspergilloma	Aspergillus empyema	*Aspergillus*	Irrigation and debridement of empyema	Recovered
3	van Ede et al. [8]	29/M	Acute myelogenous leukemia	Pulmonary invasive aspergillosis	*Aspergillus *	Amphotericin B	Died
4	Müller et al. [9]	40/M	Chronic myelogenous leukemia	Progressive dyspnea, lungs consolidation	*Aspergillus*	Amphotericin B	Died
5	Owens et al. [10]	14/M	Acute lymphoblastic leukemia	Pulmonary nodules	*Aspergillus *	Amphotericin B	Recovered
6	Yilmaz et al. [11]	59/F	Acute lymphoblastic leukemia	Invasive pulmonary aspergillosis	*Aspergillus *	Amphotericin B	Recovered
7	Serrano-Gonzalez et al. [12]	5/M	Acute lymphoblastic leukemia	Invasive pulmonary aspergillosis	*Aspergillus *	Amphotericin B, itraconazole	Recovered
8	Bhindi and Rees [13]	92/M	Large cell esophageal lymphoma	Pneumopericardium	*Candida albicans*	Fluconazole	Recovered
9	Li et al. [14]	60/M	N/A	Pericarditis	*Candida glabrata*, *K. pneumoniae*, strep. viridans	Antimycotics, antibiotics	Recovered
